# COVID-19 Therapy: Could a Chlorophyll Derivative Promote Cellular Accumulation of Zn^2+^ Ions to Inhibit SARS-CoV-2 RNA Synthesis?

**DOI:** 10.3389/fpls.2020.01270

**Published:** 2020-08-14

**Authors:** Nicole F. Clark, Andrew W. Taylor-Robinson

**Affiliations:** ^1^Institute for Applied Ecology, University of Canberra, Bruce, ACT, Australia; ^2^Infectious Diseases Research Group, School of Health, Medical & Applied Sciences, Central Queensland University, Brisbane, QLD, Australia

**Keywords:** COVID-19, SARS-CoV-2, therapy, chlorophyll, tetrapyrrole, zinc pheophorbide, cytotoxicity, RNA synthesis

## Medicinal Uses of Chlorophyll Derivatives

A range of tetrapyrrole derivatives are in development for their observed cytotoxicity (Kang et al., 2018; Singh et al., 2019). Non-toxic, water-soluble chlorophyll derivative compounds are a popular dietary supplement among health-conscious members of high-income countries. Many organometallic compounds derived from chlorophyll *a* or *b* are approved for human consumption. For instance, sodium copper chlorophyllin is promoted for both its anti-bacterial and anti-viral properties (Ulbricht et al., 2014; Solymosi and Mysliwa-Kurdziel, 2017). It contains replacement ions of Cu^2+^ instead of Mg^2+^, which aids its water solubility and intestinal absorption (Ferruzzi and Blakeslee, 2007; Mishra et al., 2011; Ulbricht et al., 2014). While most derivatives are not deemed suitable for non-prescribed ingestion, their use in the medical field, particularly for the treatment of cancers, is considered a far safer alternative to most conventional therapies (Solymosi and Mysliwa-Kurdziel, 2017). Intramuscular injection and intravenous infusion are common modes of drug delivery for compounds derived from chlorophyll *a* in a clinical setting (Yoshida et al., 1980; Wang et al., 2001; Kochneva et al., 2010; Ulbricht et al., 2014).

## Chlorophyll Derivatives and Free Zn^2+^

Zinc pheophorbide *a* (ZnPh), a chlorophyll derivative for which a Zn^2+^ ion replaces the naturally occurring Mg^2+^, is similar to non-metallated organic molecules in possessing photosensitiser properties. These are harnessed to produce a cytotoxic effect in the treatment of cancer cells (Ahn et al., 2012; Jakubowska et al., 2013; Ocakoglu et al., 2015; Ahn et al., 2017; Solymosi and Mysliwa-Kurdziel, 2017; Mokwena et al., 2018; Singh et al., 2019). While this is particularly relevant to human adenocarcinoma cell lines, the most commonly used *in vitro* model to represent the alveoli of the lungs, a broad array of cell types has been shown to respond to ZnPh (Jakubowska et al., 2013; Ocakoglu et al., 2015). Zn^2+^ ions attached to this tetrapyrrole derivative can pass through cell membranes as it is water-soluble. Aggregation and accumulation by different organelles of pheophorbide *a* and other chlorophyll breakdown products, both with and without Zn^2+^, are demonstrated in many studies (Tao et al., 1990; Szczygieł et al., 2008; Yoon et al., 2011; Ahn et al., 2012; Tamiaki et al., 2012; Xodo et al., 2012; Jakubowska et al., 2013; Ocakoglu et al., 2015; Ahn et al., 2017; Kang et al., 2018; Mokwena et al., 2018; Singh et al., 2019; Zhou et al., 2019; Tamiaki et al., 2020). Interestingly, no localization of ZnPh to mitochondria has been observed (Jakubowska et al., 2013; Ocakoglu et al., 2015). Therefore, ZnPh is unlikely to impair the function of mitochondria in healthy cells, highlighting a number of potential benefits to this treatment. Moreover, since the charge separation of Cu^2+^ and Zn^2+^ ions is identical, with the latter displaying only a slightly higher electronegativity, ion substitution of pheophorbides with Zn^2+^ aids the solubility of ZnPh. Hence, this improves the ability of ZnPh to be a carrier molecule for the free ionisation of Zn^2+^ (Ferruzzi and Blakeslee, 2007; Jakubowska et al., 2013; Ocakoglu et al., 2015; Martinez De Pinillos Bayona et al., 2017; Kang et al., 2018) and thus potentially to act as a drug delivery system.

It has been suggested that as a clinical treatment, ZnPh may be used in conjunction with photodynamic therapy (ultraviolet B irradiation) to induce singlet oxygen stress and ionisation of Zn^2+^ molecules, resulting in cytotoxicity towards carcinogenic cells lining the human lungs (Jakubowska et al., 2013). There is also a low overall cytotoxic effect in murine models, with animals generally healthy throughout treatment indicative of limited systemic side effects (Jakubowska et al., 2013; Ocakoglu et al., 2015). As with non-metallated organic molecules, above a threshold concentration ZnPh, is cytotoxic in the dark, a phenomenon that continues upon illumination; however, at low concentrations, no cytotoxic effect is observed regardless of the level of light intensity (Jakubowska et al., 2013; Ocakoglu et al., 2015). Exposure and singlet oxygen stress can be controlled according to light spectrum settings, i.e. red light versus blue light. Whether irradiated or not, light-induced cytotoxicity that is triggered by pheophorbides is dose-related and diminishes rapidly (Ahn et al., 2012; Jakubowska et al., 2013; Ocakoglu et al., 2015), and no systemic toxicity has been detected (Ahn et al., 2017).

ZnPh and similar non-metallated forms facilitate the successful transmembrane passage of non-polar components of a molecule, wherein irradiance alters the ionic concentration of free ions once it has entered into the cytoplasm (Martinez De Pinillos Bayona et al., 2017). Free Zn^2+^ appears to promote an antiviral effect, research *in vitro* demonstrating accumulation of Zn^2+^ in human lung tissue (Hagimori et al., 2019). Moreover, increasing the cellular concentration of Zn^2+^ through zinc supplementation and using ionophores such as pyrithione efficiently impairs RNA replication by human coronaviruses, leading to improved treatment outcomes (te Velthuis et al., 2010; Read et al., 2019; Derwand and Scholz, 2020; Zhang and Liu, 2020). Similarly, sucking zinc lozenges reduces symptoms of viral respiratory infection (Turner and Cetnarowski, 2000). Zinc ionophores are effective at increasing concentrations of Zn^2+^ because they allow more zinc to pass through the cell membrane (Zhang and Liu, 2020). Yet, as uptake of zinc is a less efficient and therefore slower metabolic process in eukaryotes (Krężel and Maret, 2006), very little is known about zinc ionophores as transporters (Gaither and Eide, 2001).

The primary function of an ionophore is to increase cell permeability to enable transport of ionic compounds across the cell membrane by endocytosis. Although this is a role fundamental to normal functioning of a healthy cell, the presence of ionophores can weaken the integrity of the cell membrane, thus paradoxically leading to decreased cell defence systems (Gaither and Eide, 2001; te Velthuis et al., 2010; Derwand and Scholz, 2020). However, it is not known if ZnPh also weakens cell membranes nor whether the rate and/or extent of ionisation of free zinc is increased during this uptake. Despite ZnPh not being a recognized ionophore, as it is highly soluble when irradiated, uptake of Zn^2+^ is likely to be improved by this process (Szczygieł et al., 2008; Tamiaki et al., 2012; Martinez De Pinillos Bayona et al., 2017; Hagimori et al., 2019). Studies using *in vitro* cultured cells point to the capacity of ZnPh to elevate free ion concentrations of Zn^2+^ (Jakubowska et al., 2013; Ocakoglu et al., 2015). If this effect translates successfully from *in vitro* to *in vivo*, then such a mechanism would reduce the therapeutic demand for the use of toxic or dose-dependent ionophores such as hydroxychloroquine.

## Clinical Capacity and Therapeutic Potential of Zinc Pheophorbide *a*

While our understanding of the metabolic processes of zinc transporters remains limited, it is plausible to speculate that ZnPh combined with zinc supplementation could reach deep inside human lungs (Jakubowska et al., 2013). This would increase the cellular concentration of Zn^2+^ to target cells such as alveolar epithelia (Hagimori et al., 2019), corroborating *in vitro* studies on the free ionisation of Zn^2+^ (Szczygieł et al., 2008; Tamiaki et al., 2012; Hagimori et al., 2019; Zhou et al., 2019). Additionally, such raised intracellular levels may be achieved independently of the need for a cytotoxic effect experienced both in the dark and upon illumination, eliminating associated risks of singlet oxygen stress, ionisation and phosphorylated nuclear cell damage. Therefore, since ZnPh is water-soluble, it is reasonable to consider that ionic uptake of Zn^2+^ is increased, potentially producing a similar effect as observed with other ionophores such as pyrithione and hydroxychloroquine (Derwand and Scholz, 2020). Together with zinc supplementation, this may significantly impair replication of human coronaviruses, notably SARS-CoV-2, under experimental conditions. Hence, we propose that this novel therapy merits further evaluation to develop long-term as a possible clinical treatment of symptomatic COVID-19 patients.

The drug delivery capacity of chlorophyll derivatives depends on the properties of each compound (Ulbricht et al., 2014; Kang et al., 2018), as some display non-polar behaviour in a dose-dependent ratio (Jakubowska et al., 2013; Ocakoglu et al., 2015; Martinez De Pinillos Bayona et al., 2017), thus emphasising the need for clinical trials (Tang et al., 2006; Ferruzzi and Blakeslee, 2007; Mishra et al., 2011; Ulbricht et al., 2014; Solymosi and Mysliwa-Kurdziel, 2017). It is important to appreciate that replacement of the central ion in pheophorbides can yield varied results *in vitro* and *in vivo*, with some studies suggesting that ionic inclusion may reduce toxicity (Szczygieł et al., 2008; Tamiaki et al., 2012; Martinez De Pinillos Bayona et al., 2017; Tamiaki et al., 2020). Accidental ingestion of non-dietary levels of pheophorbide *a*, a compound that lacks a central ion, induces a rare but mild phototoxic effect at low doses; however, this was still deemed safe for human consumption (Hwang et al., 2005).

Chlorophyll is generally considered not harmful to humans (Ulbricht et al., 2014), but information is scarce on any possible toxicity posed by chlorophyll derivatives, especially those compounds, both with and without ion inclusion, e.g. Zn^2+^, which have not been subject to clinical trials. Pre-clinical and clinical safety tests on ZnPh are required. However, it is generally accepted that toxicity is dependent on a photo-reactive response (Tao et al., 1990; Hwang et al., 2005; Szczygieł et al., 2008; Tamiaki et al., 2012; Jakubowska et al., 2013; Ocakoglu et al., 2015; Martinez De Pinillos Bayona et al., 2017; Zhou et al., 2019; Tamiaki et al., 2020), given that phototoxicity induced by ZnPh is short-lived (Ahn et al., 2012; Jakubowska et al., 2013; Ocakoglu et al., 2015; Solymosi and Mysliwa-Kurdziel, 2017). Rapid clearance of zinc chlorophyll compounds is observed in human cell lines, with low doses invoking no or limited cytotoxic effect (Tao et al., 1990; Tang et al., 2006; Jakubowska et al., 2013; Ocakoglu et al., 2015; Martinez De Pinillos Bayona et al., 2017; Diogo et al., 2018).

## Conclusion

Here, we provide an insight into the properties of ZnPh that make this chlorophyll derivative a potential therapeutic agent for treating COVID-19. Our understanding of ZnPh and similar compounds relates to the reported success of *in vitro* and *in vivo* cancer studies, so this recognised drug delivery system has not been used yet as a treatment for respiratory illness. However, the possibility that this may translate to an anti-viral property merits investigation.

Unless a pronounced phototoxic effect is induced — highly unlikely under physiological conditions — ZnPh is non-toxic to humans. Therefore, there is a strong likelihood that this compound offers great potential to act as a carrier molecule for Zn^2+^ to trigger an anti-viral response that impairs SARS-CoV-2 replication. Hence, Zn^2+^ ionophores provide a novel therapeutic option that may be of benefit to the development of an effective treatment for the severe clinical manifestations of COVID-19.

## Author Contributions

Both authors (NC and AT-R) made substantial contributions to the conception of the work and to literature search, contributed significantly to writing the manuscript, revised it critically for important intellectual content, approved its final version and agreed to its submission.

## Conflict of Interest

The authors declare that the research was conducted in the absence of any commercial or financial relationships that could be construed as a potential conflict of interest.

## References

[B1] Ahn M.-Y. Kwon S.-M. Kim Y.-C. Ahn S.-G. Yoon J.-H. (2012). Pheophorbide *a*-mediated photodynamic therapy induces apoptotic cell death in murine oral squamous cell carcinoma *in vitro* and *in vivo*. Oncol. Rep. 27, 1772–1778. 10.3892/or.2012.1748 22470106

[B2] Ahn M.-Y. Yoon H.-E. Moon S.-Y. Kim Y.-C. Yoon J.-H. (2017). Intratumoral photodynamic therapy with newly synthesized pheophorbide a in murine oral cancer. Oncol. Res. 25, 295–304. 10.3727/096504016X14732527645922 27629775 PMC7841246

[B3] Derwand R. Scholz M. (2020). Does zinc supplementation enhance the clinical efficacy of chloroquine/hydroxychloroquine to win todays battle against COVID-19? Med. Hypotheses 142, 109815. 10.1016/j.mehy.2020.109815 32408070 PMC7202847

[B4] Diogo P. Mota M. Fernandes C. Sequeira D. Palma P. Caramelo F. (2018). Is the chlorophyll derivative Zn(II)e_6_Me a good photosensitizer to be used in root canal disinfection? Photodiagn. Photodyn. Ther. 22, 205–211. 10.1016/j.pdpdt.2018.04.009 29684689

[B5] Ferruzzi M. G. Blakeslee J. (2007). Digestion, absorption, and cancer preventative activity of dietary chlorophyll derivatives. Nutr. Res. 27, 1–2. 10.1016/j.nutres.2006.12.003

[B6] Gaither L. A. Eide D. J. (2001). Eukaryotic zinc transporters and their regulation. Biometals 14, 251–270. 10.1023/A:1012988914300 11831460

[B7] Hagimori M. Taniura M. Mizuyama N. Karimine Y. Kawakami S. Saji H. (2019). Synthesis of a novel pyrazine–pyridone biheteroaryl-based fluorescence sensor and detection of endogenous labile zinc ions in lung cancer cells. Sensors 19, 2049. 10.3390/s19092049 PMC6540122 31052519

[B8] Hwang D. F. Tsai Y. S. Chou S. S. Liu S. M. Wu J. T. Lin S. J. (2005). HPLC determination of pheophorbide *a* and pyropheophorbide *a* in dried laver product implicated in food poisoning. Shokuhin Eiseigaku Zasshi: J. Food Hyg. Soc. Jpn. 46, 45–48. 10.3358/shokueishi.46.45 16018590

[B9] Jakubowska M. Szczygieł M. Michalczyk-Wetula D. Susz A. Stochel G. Elas M. (2013). Zinc-pheophorbide *a* — highly efficient low-cost photosensitizer against human adenocarcinoma in cellular and animal models. Photodiagn. Photodyn. Ther. 10, 266–277. 10.1016/j.pdpdt.2012.12.004 23993853

[B10] Kang Y.-R. Park J. Jung S. K. Chang Y. H. (2018). Synthesis, characterization, and functional properties of chlorophylls, pheophytins, and Zn-pheophytins. Food Chem. 245, 943–950. 10.1016/j.foodchem.2017.11.079 29287463

[B11] Kochneva E. V. Filonenko E. V. Vakulovskaya E. G. Scherbakova E. G. Seliverstov O. V. Markichev N. A. (2010). Photosensitizer Radachlorin®: Skin cancer PDT phase II clinical trials. Photodiagn. Photodyn. Ther. 7, 258–267. 10.1016/j.pdpdt.2010.07.006 21112549

[B12] Krężel A. Maret W. (2006). Zinc-buffering capacity of a eukaryotic cell at physiological pZn. J. Biol. Inorg. Chem. 11, 1049–1062. 10.1007/s00775-006-0150-5 16924557

[B13] Martinez De Pinillos Bayona A. Mroz P. Thunshelle C. Hamblin M. R. (2017). Design features for optimization of tetrapyrrole macrocycles as antimicrobial and anticancer photosensitizers. Chem. Biol. Drug Des. 89, 192–206. 10.1111/cbdd.12792 28205400 PMC5319686

[B14] Mishra V. K. Bacheti R. K. Husen A. (2011). “Medicinal uses of chlorophyll: a critical overview,” in Chlorophyll: Structure, Function and Medicinal Uses. Eds. Le H. Salcedo E. (Hauppauge, NY: Nova Scientific), 177–196.

[B15] Mokwena M. G. Kruger C. A. Ivan M.-T. Heidi A. (2018). A review of nanoparticle photosensitizer drug delivery uptake systems for photodynamic treatment of lung cancer. Photodiagn. Photodyn. Ther. 22, 147–154. 10.1016/j.pdpdt.2018.03.006 29588217

[B16] Ocakoglu K. Er O. Kiyak G. Lambrecht F. Y. Gunduz C. Kayabasi C. (2015). 131I–Zn–chlorophyll derivative photosensitizer for tumor imaging and photodynamic therapy. Int. J. Pharm. 493, 96–101. 10.1016/j.ijpharm.2015.07.047 26226337

[B17] Read S. A. Obeid S. Ahlenstiel C. Ahlenstiel G. (2019). The role of zinc in antiviral immunity. Adv. Nutr. 10, 696–710. 10.1093/advances/nmz013 31305906 PMC6628855

[B18] Singh K. Chaturvedi D. Singh V. K. (2019). Review on different aspects of the photodynamic product pheophorbide. Spec. J. Biol. Sci. 5, 6–13.

[B19] Solymosi K. Mysliwa-Kurdziel B. (2017). Chlorophylls and their derivatives used in food industry and medicine. Mini Rev. Med. Chem. 17, 1194–1222. 10.2174/1389557516666161004161411 27719668

[B20] Szczygieł M. Urbańska K. Jurecka P. Stawoska I. Stochel G. Fiedor L. (2008). Central metal determines pharmacokinetics of chlorophyll-derived xenobiotics. J. Med. Chem. 51, 4412–4418. 10.1021/jm7016368 18605716

[B21] Tamiaki H. Okamoto Y. Mikata Y. Shoji S. (2012). Photooxidative cleavage of zinc 20-substituted chlorophyll derivatives: conformationally *P*-helix-favored formation of regioselectively 19–20 opened linear tetrapyrroles. Photochem. Photobiol. Sci. 11, 898–907. 10.1039/C1PP05301A 22139399

[B22] Tamiaki H. Fukai K. Nakamura S. (2020). Intramolecular interaction of synthetic chlorophyll heterodyads with different π-skeletons. Photochem. Photobiol. Sci. 19, 332–340. 10.1039/C9PP00373H 31994582

[B23] Tang P. M.-K. Chan J. Y.-W. Au S. W.-N. Kong S.-K. Tsui S. K.-W. Waye M. M.-Y. (2006). Pheophorbide *a*, an active compound isolated from *Scutellaria barbata*, possesses photodynamic activities by inducing apoptosis in human hepatocellular carcinoma. Cancer Biol. Ther. 5, 1111–1116. 10.4161/cbt.5.9.2950 16880732

[B24] Tao H. Sun Z. Liu M. Wen X. (1990). Synthesis of zinc chlorophyllin *a* and its preliminary clinical application. Hua Xi Yi Ke Da Xue Xue Bao 21, 341–343. 2093078

[B25] te Velthuis A. J. W. van den Worm S. H. Sims A. C. Baric R. S. Snijder E. J. van Hemert M. J. (2010). Zn^2+^ inhibits coronavirus and arterivirus RNA polymerase activity *in vitro* and zinc ionophores block the replication of these viruses in cell culture. PLoS Pathog. 6, e1001176. 10.1371/journal.ppat.1001176 21079686 PMC2973827

[B26] Turner R. B. Cetnarowski W. E. (2000). Effect of treatment with zinc gluconate or zinc acetate on experimental and natural colds. Clin. Infect. Dis. 31, 1202–1208. 10.1086/317437 11073753

[B27] Ulbricht C. Bramwell R. Catapang M. Giese N. Isaac R. Le T. D. (2014). An evidence-based systematic review of chlorophyll by the Natural Standard Research Collaboration. J. Diet. Suppl. 11, 198–239. 10.3109/19390211.2013.859853 24670123

[B28] Wang H. Tan Y. Wang X. Xie J. (2001). Identification of pelvic lymph nodes with chlorophyllin after injection into the uterine cervix: an experimental and clinical study. Lymphology 34, 69–76. 11471574

[B29] Xodo L. E. Rapozzi V. Zacchigna M. Drioli S. Zorzet S. (2012). The chlorophyll catabolite pheophorbide *a* as a photosensitizer for the photodynamic therapy. Curr. Med. Chem. 19, 799–807. 10.2174/092986712799034879 22214455

[B30] Yoon I. Park H.-S. Cui B. C. Li J. Z. Kim J.-H. Lkhagvadulam B. (2011). Photodynamic and antioxidant activities of divalent transition metal complexes of methyl pheophorbide-*a*. Bull. Korean Chem. Soc 32, 2981–2987. 10.5012/bkcs.2011.32.8.2981

[B31] Yoshida A. Yokono O. Oda T. (1980). Therapeutic effect of chlorophyll-*a* in the treatment of patients with chronic pancreatitis. Gastroenterol. Jpn. 15, 49–61. 10.1007/BF02773704 6153629

[B32] Zhang L. Liu Y. (2020). Potential interventions for novel coronavirus in China: a systemic review. J. Med. Virol. 92, 479–490. 10.1002/jmv.25707 32052466 PMC7166986

[B33] Zhou H. Xia L. Zhong J. Xiong S. Yi X. Chen L. (2019). Plant-derived chlorophyll derivative loaded liposomes for tri-model imaging guided photodynamic therapy. Nanoscale 11, 19823–19831. 10.1039/C9NR06941K 31633141

